# Pollen Killer Gene *S35* Function Requires Interaction with an Activator That Maps Close to *S24*, Another Pollen Killer Gene in Rice

**DOI:** 10.1534/g3.116.027573

**Published:** 2016-03-21

**Authors:** Takahiko Kubo, Atsushi Yoshimura, Nori Kurata

**Affiliations:** * Plant Genetics Laboratory, National Institute of Genetics, Mishima, Shizuoka 411-8540, Japan; † Department of Genetics, Graduate University for Advanced Science (SOKENDAI), Mishima, Shizuoka 411-8540, Japan; ‡ Plant Breeding Laboratory, Division of Genetics and Plant Breeding, Department of Applied Genetics and Pest Management, Faculty of Agriculture, Kyushu University, Fukuoka 812-8581, Japan

**Keywords:** pollen killer, *Oryza sativa*, epistasis, reproductive isolation

## Abstract

Pollen killer genes disable noncarrier pollens, and are responsible for male sterility and segregation distortion in hybrid populations of distantly related plant species. The genetic networks and the molecular mechanisms underlying the pollen killer system remain largely unknown. Two pollen killer genes, *S24* and *S35*, have been found in an intersubspecific cross of *Oryza sativa* ssp. *indica* and *japonica*. The effect of *S24* is counteracted by an unlinked locus *EFS*. Additionally, *S35* has been proposed to interact with *S24* to induce pollen sterility. These genetic interactions are suggestive of a single *S24*-centric genetic pathway (*EFS–S24–S35*) for the pollen killer system. To examine this hypothetical genetic pathway, the *S35* and the *S24* regions were further characterized and genetically dissected in this study. Our results indicated that *S35* causes pollen sterility independently of both the *EFS* and *S24* genes, but is dependent on a novel gene close to the *S24* locus, named *incentive for killing pollen* (*INK*). We confirmed the phenotypic effect of the *INK* gene separately from the *S24* gene, and identified the *INK* locus within an interval of less than 0.6 Mb on rice chromosome 5. This study characterized the genetic effect of the two independent genetic pathways of *INK–S35* and *EFS–S24* in *indica–japonica* hybrid progeny. Our results provide clear evidence that hybrid male sterility in rice is caused by several pollen killer networks with multiple factors positively and negatively regulating pollen killer genes.

Hybrid progeny derived from genetically divergent species frequently suffer reproductive dysfunction. This phenomenon, referred to as hybrid sterility, is assumed to play an important role in the development of speciation by triggering postzygotic reproductive isolation. Segregation distorters, also called transmission ratio distortion factors, are evolutionarily selfish genetic elements that distort Mendelian segregation in their favor at the expense of others. Segregation distorters have been reported in a wide variety of reproductive organisms, including animals, plants, and fungi (Burt and Trivers 2009), and are one of the primary origins of hybrid sterility by causing one-half of the gametes to be dysfunctional. Two well-characterized genetic elements are *Segregation distorter* (*Sd*) in *Drosophila* (Larracuente and Presgraves 2012) and *t*-complex in mice (Lyon 2003). The role of segregation distorters in speciation of reproductive organisms has been the subject of considerable discussion (Burt and Trivers 2009).

In plants, diverse types of segregation distorters have been identified in many plant species. Some examples include: pollen killer in *Nicotiana* (Cameron and Moav 1957), gamete eliminator in tomato (Rick 1966), gametocidal factor in wheat (Endo 2015), chromosomal knobs in maize (Buckler *et al.* 1999; Kanizay *et al.* 2013) and female meiotic drive in *Mimulus* (Fishman *et al.*and Saunders 2008; Finseth *et al.* 2015). *Gamete eliminator* (*Ge*) (both pollen and egg are abortive) was found in a tomato intraspecific cross by plant geneticist Charles M. Rick, who interpreted *Ge* as a triallelic system at a single locus (Rick 1966). The gamete eliminator (*Ge^p^*) causes abortion of gametes carrying *Ge^c^* in the *Ge^p^/Ge^c^* heterozygote. The third neutral allele, *Ge^n^*, is compatible with both *Ge^p^* and *Ge^c^* in the heterozygous hybrid. Since the neutral allele *Ge^n^* is widely distributed, Rick proposed that “both *Ge^c^* and *Ge^p^* could arise by mutation from *Ge^n^* without adverse effect on gamete fertility” (Rick 1966). Based on this idea, the killer and the abortive allele were proposed to be derived from a common ancestral allele that is compatible with both of them.

The egg killer locus in rice, *S5*, is a type of triallelic system (Ikehashi and Araki 1986). The killer allele *S5-i* from *indica* causes female gamete abortion in the heterozygous state with the *japonica* allele, but not with the neutral alleles from other varieties or the wild progenitor. Closer study of the *S5* system unveiled the molecular mechanism. The *S5* locus is composed of three tightly linked genes, and particular combinations of the alleles lead to endoplasmic reticulum stress, resulting in female gamete abortion (Yang *et al.* 2012). As a case study of male sterility, two adjacent genes that encode a SUMO E3 ligase-like protein and an F-box protein were identified in an *indica/japonica* cross (Long *et al.* 2008). These pioneering studies have provided important insights into the genetic architecture and the evolutionary history of plant gamete killers; namely, the existence of tightly linked multiple genes, and cumulative mutations in these genes lead to the gamete killing phenotype in heterozygotes. In this scenario, not only the ancestral haplotype, but also some of the derived haplotypes can work as compatible (neutral) haplotypes. In this context, several questions about segregation distorters remain to be explored. (1) What is a common or unique aspect of the molecular mechanism? Previous studies have identified highly repetitive and heterochromatic sequences as common causal molecules, *e.g.*, the *D* locus in *Mimulus* (Fishman and Saunders 2008), knobs in maize (Buckler *et al.* 1999), and *HSR* in mice (Weichenhan *et al.* 2001). (2) What are the normal functions of these genes within the parental species? (3) Why have a greater number of segregation distorters evolved in plant genomes compared with animal or fungal genomes? (4) Has domestication facilitated the development of segregation distorters? There are many segregation distorters found in crop species, especially in rice, and a previous study proposed an association between reproductive isolation and domestication (Dempewolf *et al.* 2012). Further studies extended to many more examples are needed to answer these questions.

Asian cultivated rice (*Oryza sativa* L.) is an autogamous diploid species (2*n* = 24) and has two major subspecies, *indica* and *japonica*, whose genomes have been sequenced (Yu *et al.* 2002; International Rice Genome Sequencing Project 2005). These subspecies are thought to have originated independently from different subpopulations of the closest wild relative *O. rufipogon* (Morishima 2001; Zhu and Ge 2005; Londo *et al.* 2006; Kovach *et al.* 2007). The intersubspecific cross often exhibits hybrid sterility due to abnormal growth of pollen and/or the embryo sac. Many cases of hybrid sterility in rice are caused by so-called “allelic interaction at a single genetic locus” rather than by epistatic interactions between unlinked loci (Koide *et al.* 2008; Ouyang *et al.* 2009). A hybrid male sterility gene, *S24*, also called *f5*-Du (Wang *et al.* 2006) or *S-b* (Li *et al.* 2006), is a typical gene showing an allelic interaction effect that is located on rice chromosome 5. *S24* causes pollen semisterility when the *indica* allele is introgressed into the *japonica* background. The *indica* allele of (*S24-i*) acts as a pollen killer and produces maldeveloped male gametes bearing the *japonica* allele exclusively in the heterozygotes (Kubo *et al.* 2008; Zhao *et al.* 2011). Consequently, the *S24-i* allele is preferentially transmitted to the offspring (90–99%). Zhao *et al.* (2011) identified an ankyrin protein gene as the primary candidate for *S24* by a fine-mapping approach. *S24* is under the control of an unlinked dominant suppressor, *Epistatic Factor for S24* (*EFS*), located on chromosome 2 (Kubo *et al.* 2011). Another hybrid male sterility gene, *S35*, located on chromosome 1, acts as a pollen killer in a similar fashion to *S24*. Interestingly, *S35* has been proposed to interact genetically with *S24* to cause pollen sterility, implying a killer–killer interaction (Kubo *et al.* 2008). Together with the animal examples, it is plausible that linked and unlinked epistatic factors play key roles in the genetic mechanism of the gamete killer system.

In contrast to the *S24* locus, which has been somewhat characterized, many characteristics of the *S35* locus remain unknown. For example, the causal gene and molecular mechanism of *S35* have not been elucidated, although the basic genetic characteristics, and the approximate position of the *S35* locus on chromosome 1 have been identified (Kubo *et al.* 2008). Also, it remains unclear whether the *S24*-suppressor gene *EFS* can restore pollen sterility due to *S35*, and whether such a killer–killer (*S24–S35*) interaction really exists as previously proposed. The aim of this study was to characterize the *S35* gene and its related genetic network more completely. Using reciprocal near-isogenic lines for *S24* and *S35*, our results indicated that the mechanism for hybrid male sterility in rice involves multiple genes and more complex gene interactions than expected. Here, we report a novel gene that interacts with *S35*, and evidence for the genetic independency of the *S24* and *S35* genes.

## Materials and Methods

### Plant materials

All experimental populations, including mapping populations and near-isogenic lines (NILs), were derived from reciprocal chromosome segment substitution lines (CSSLs), and sister lines that were obtained from a cross between the *japonica* rice variety Asominori and the *indica* variety IR24 (Kubo *et al.* 2002). Both of the parents produced fertile pollen (> 90%) under our growth conditions. An overall view of the breeding procedure used to develop the experimental populations is illustrated in Supplemental Material, Figure S1. The NILs with the Asominori genetic background were developed from crossed/backcrossed and selfed progeny of AIS 86, a CSSL carrying IR24 segments of chromosomes 1 and 5 (Kubo *et al.* 2002), if not otherwise specified. To evaluate pollen fertility of all 27 genotype classes generated by different combinations of the *S24*, *S35*, and *EFS* alleles, a triple heterozygous plant for *S24*, *S35*, and *EFS* in the Asominori genetic background (NIL-S24+S35+EFS) was developed by crossing CSSL AIS 86 and AB2-6 (a derivative line of the AIS library) (Figure S1A). The NIL S24ILH/I, carrying a very small IR24 segment around the *S24* locus in an otherwise uniform Asominori genetic background, was developed by two additional backcrosses of AIS 86 with Asominori (BC_5_F_2_) and marker-assisted selection (MAS) (Figure S1B). NILs with the IR24 genetic background were developed from crosses of three different CSSLs, IAS 27/IAS 10//IAS 6 (Figure S1C). Fine mapping of *S35* and an *S35*-activator *INK* was performed using self-pollinated progeny of AIS 86/Aso and NIL-S24+S35+EFS/Aso. Genotype frequencies were determined using seedling leaves of self-pollinated progeny of a single plant heterozygous for *S35* or *S24* with different genetic backgrounds. All plant materials for phenotype evaluation were grown under paddy field conditions in 2012–2015 in Mishima, Japan.

### DNA analysis

Crude DNA extracts from individual leaves were prepared for genotyping using 0.25 M, NaOH followed by neutralization with 0.1 M Tris-HCl. These DNA extracts (1.0 µl) were used in PCR reactions (10 µl final volume) performed using GoTaq polymerase (Promega, Fitchburg, WI) with the following cycling profile: 94° for 2 min; followed by 30 cycles of 94° for 20 sec, 50−60° for 20 sec, and 72° for 30 sec. The PCR products were resolved on 2.0% agarose gels, and visualized by ethidium bromide staining. PCR-based markers, insertion and deletion (InDel), and simple sequence repeat (SSR) markers were newly designed for mapping of the *S35* and *INK* loci based on DNA sequence polymorphisms between Nipponbare and 93-11 (MSU6, http://rice.plantbiology.msu.edu/; BGI, http://rice.genomics.org.cn/rice/index2.jsp). Also, SSR markers reported by McCouch *et al.* (2002) were used. The primer sequences for DNA markers are listed in Table S1. The whole-genome genotyping data of the reciprocal CSSLs were obtained from Kubo *et al.* (2002). For genotyping nontarget chromosome segments retained in the genetic background, PCR markers were used that were evenly distributed over the 12 rice chromosomes (Mizuta *et al.* 2010; Kubo *et al.* 2011). Genes and marker loci are shown on the physical chromosome map based on the Nipponbare sequence (MSU7).

### Phenotyping and histological experiments

Preflowering panicles from each individual in the population were collected and fixed in 50% ethanol solutions to examine pollen fertility. Pollen grains from three to six anthers at 1 d before anthesis were stained with 1.0% iodine-potassium iodide (I_2_-KI), and the number of stained/unstained pollen grains were counted using a microscope. More than 400 pollen grains were scored for each individual. In this study, we scored pollen phenotypes with <40%, 40–70%, 70–90%, and >90% pollen fertility as sterile, semisterile, partial sterile, and fertile, respectively. To observe the morphology of mature pollen grains, the ethanol-fixed pollen grains were stained with hematoxylin solution by the method of Kindiger and Beckett (1985). Male gametogenesis was analyzed using young panicles at different developmental stages collected from Asominori and the pollen sterile plants. The panicles were fixed and stored in FAA solution (45% ethanol, 5% formalin, and 5% acetic acid). After fixation, the samples were embedded in paraffin (Paraplast Plus; McMormick Scientific, St. Louis, MO) and cut at a thickness of 8 µm with a Microm HM355 microtome (Microm, Walldorf, Germany). The sections were stained with 0.05% Toluidine blue O. To evaluate seed fertility, three panicles with fully ripened grains were collected from each plant, and the numbers of filled and unfilled spikelets were counted. The seed setting rate was estimated by the formula: the number of filled grains / total number of filled and unfilled grains.

### Data availability

The authors state that all data necessary for confirming the conclusions presented in the article are represented fully within the article.

## Results

### Characteristics and chromosomal localization of S35

**Figure 1:**
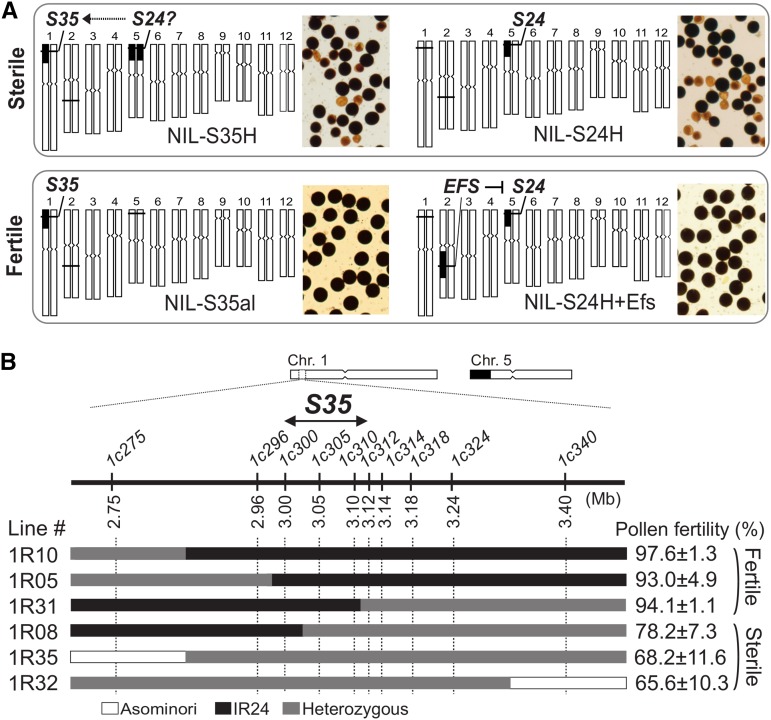
Genetic basis of hybrid male sterility genes and localization of the *S35* locus. (A) Pollen phenotypes of NILs for *S35*, *S24*, and *EFS* genes associated with hybrid male sterility. Graphical genotypes (left) and pollen grains (right) of the NILs stained by I_2_-KI are shown. Black and white bars denote IR24 (*indica*) and Asominori (*japonica*) chromosomes, respectively. The NIL carrying *S35* alone (NIL-S35al) were fertile, but cointrogression of the *S24* segment from IR24 dominantly activated *S35*, leading to pollen sterility in the Asominori genetic background (NIL-S35H). The *S24*-suppressor *Efs* gene from IR24 dominantly inactivated *S24* (NIL-S24H+Efs). (B) Chromosome position of *S35*. Upper panel: Physical chromosome positions of the *S35* locus and DNA markers (*1cXXX*). Lower panel: Diagram showing the recombination breakpoints and pollen phenotypes of the lines. All recombinant plants carried IR24 homozygous alleles for *S24* to activate *S35-i*. The pollen fertility is shown as the mean (%) ± SD (*n* = 3–7).

**Figure 2:**
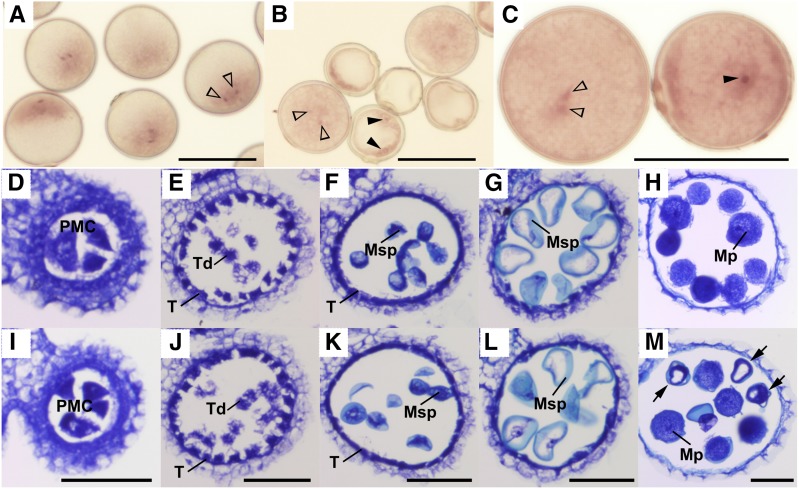
Histological characterization of the sterile *S35* heterozygotes. (A)–(C) Mature pollen grains stained with hematoxylin. (A) Pollen grains from Asominori. (B), (C) Pollen grains from the sterile *S35* heterozygotes (NIL-S35H). White and black arrowheads indicate normal sperm cells and nuclei of abortive pollen grains, respectively. Pollen grains arrested at the bicellular stage (black arrowheads) and vacuolated pollen at the uninucleate stage are shown in (B). (C) shows that these two pollen grains from NIL-S35H were well filled and similar in size, but the pollen grain on the right has a single bold nucleus instead of two sperm cells as would be observed in normal pollen grains. (D)–(M) Transverse sections showing anther development of Asominori and the *S35* heterozygote (NIL-S35H). Five stages of anther development in Asominori and NIL-S35H were compared. Transverse sections were stained with 0.05% Toluidine blue O. (D)–(H) are Asominori, and (I)–(M) are NIL-S35H. (D) and (I) the pollen mother cell stage, (E) and (J) the tetrad stage, (F) and (K) the young microspore stage, (G) and (L) vacuolated pollen stage, (H) and (M) the mature pollen stage. The solid black arrows indicate degenerated pollen grains. PMC, pollen mother cell; T, tapetal layer; Td, tetrad cell; Msp, microspore; Mp, mature pollen. Scale bars = 50 µm for (A)–(M).

The pollen killer gene *S35* has been roughly mapped to the short arm of rice chromosome 1 (Kubo *et al.* 2008). This previous genetic study indicated that the heterozygous *S35* (*S35-i/S35-j*) caused pollen sterility when the *indica* segment harboring *S24*, another pollen killer, was concurrently introgressed into the *japonica* background (NIL-S35H in Figure 1A) (Kubo *et al.* 2008). A single introgression of *S35-i* in the *japonica* genome does not cause pollen sterility (NIL-S35al in Figure 1A). These observations suggested that *S35-i* interacts with the dominant *indica* allele of *S24* (*S24-i*) (Kubo *et al.* 2008). Reciprocal test crossing of NIL-S35H and Asominori showed that *S35-i* was inherited at a higher frequency (89%) through the male gamete in the *S35* heterozygotes (the expected frequency was 50%) (Table 1), indicating the selective abortion of the male gamete bearing *S35-j*, and the incomplete penetrance of the *S35* gene. Histological observations showed that the mature anthers of the sterile NIL-S35H retained uninucleate and bicellular microspores, suggesting that developmental defects begin from the late uninucleate microspore stage after successful meiosis (Figure 2, A and B). Pollen grains arrested at later developmental stages were also found (Figure 2C). This finding suggested that evaluating some of the large arrested pollen grains as “fertile pollen” might have resulted in an overestimation of pollen fertility in the *S35* heterozygotes. No apparent defects were found in the tapetum or anther walls of sterile *S35*-heterozygotes during male gametogenesis (Figure 2, D–L). We next performed high-resolution mapping to determine the precise position of the *S35* locus. Using a large segregating population (*N* = 2800), the location of the *S35* locus was narrowed down within a 122-kb region between marker loci *1c300–1c312* (Figure 1B). The candidate genomic region for *S35* encoded 27 putative protein-coding genes and four transposable elements (Table S2). Rice transcriptome data (Fujita *et al.* 2010) indicated that, out of the 27 candidate genes, three genes (LOC_Os01g06460/Cys-rich domain protein, LOC_Os01g06580/fasciculin-like arabinogalactan-protein, and LOC_Os01g06590/zinc finger C3HC4 type domain protein) had relatively higher expression, or their expression significantly changed around the uninucleate stage (Figure S2), suggesting that these three genes are the most probable candidates for *S35* function.

**Table 1:** Transmission frequency of *S35-i*-bearing gametes in reciprocal test crosses between NIL-S35H and Asominori

Cross Combination		No. of Plants*^a^*		Total	χ^2^ (1:1)	Frequency of the *indica* Allele (*k*_i_)*^b^*
♀	♂	*japonica*	Heterozygote			
Asominori	NIL-S35H	17	134	151	45.9***	0.89
NIL-S35H	Asominori	28	43	71	1.63^NS^	0.61

*** *P* < 0.001. NS, not significant.

a DNA marker *1c350* linked to *S35* was used for genotyping.

b The expected frequency is 0.5.

### Pollen sterility due to S35 is independent of EFS

**Figure 3:**
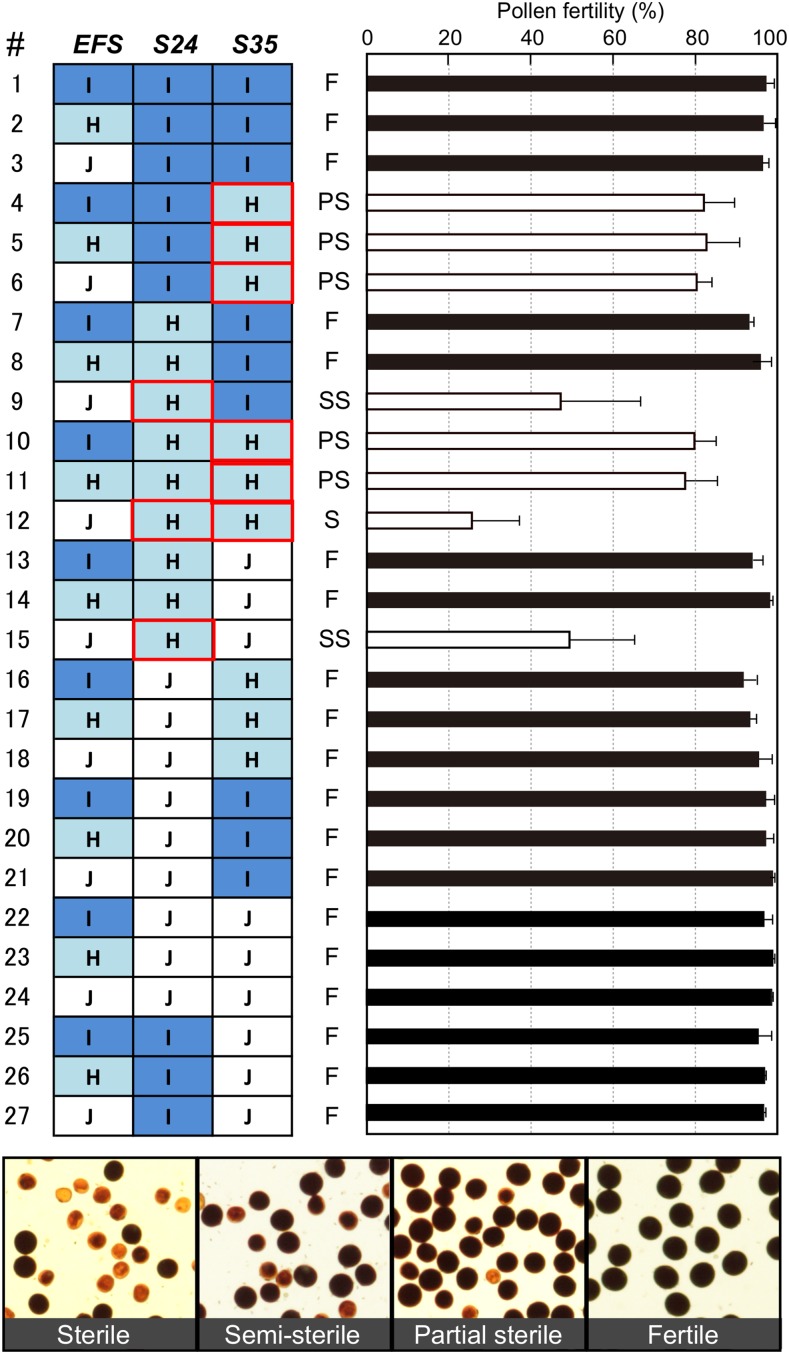
Pollen fertility of 27 genotypes determined by three genes *S24*, *S35*, and *EFS*. Pollen fertility (right bar chart) for each genotype class (left panel) is expressed by the mean (%) ± SD (*n* = 3–10). Effective genes for pollen sterility are marked with red frames on the genotype panel. White and black bars in the bar chart represent the sterile and fertile pollen phenotypes, respectively. Micrographs of the pollen grains for each fertility class are shown in the bottom panel. Plants of each genotype were selected from self-pollinated progeny of NIL-S24+S35+Efs by genotyping using the linked markers *mS2* for *S24*, *1c305* for *S35*, and *2c2015* for *EFS*. I: *indica* (IR24) homozygote. J, *japonica* (Asominori) homozygote; H, heterozygote; F, fertile; PS, partial-sterile; SS, semisterile; S, sterile.

The *EFS* locus was previously identified as a suppressor of *S24*; the dominant *indica* allele *Efs-i* restores pollen sterility due to *S24* (NIL-S24H+Efs in Figure 1A) (Kubo *et al.* 2011). A direct or indirect interaction between *EFS* and *S35* was presumed based on the previously identified genetic interactions (*S24*–*S35* and *EFS*–*S24*) (Kubo *et al.* 2008, 2011). To examine whether the *S35* and *EFS* genes interact with each other, we phenotyped all 27 allele combinations at the three loci *S24*, *EFS*, and *S35*. The self-pollinated progeny of triple heterozygous plants for *S24*, *EFS*, and *S35* were used to evaluate the 27 genotypes. Consistent with the previous results (Kubo *et al.* 2008, 2011), *S35* caused pollen sterility only in the presence of the *S24-i* allele (#6 and #18 in Figure 3), and *Efs-i* restored the *S24*-dependent pollen sterility (#13–#15 in Figure 3). However, contrary to our expectations, the heterozygous *S35* caused partial pollen sterility regardless of the *EFS* genotype (#4–#6 in Figure 3). This result indicated that *Efs-i* is unable to suppress *S35*, and, therefore, the *S24*-specific suppressor. Thus, *S35* required the IR24 segment harboring *S24* to induce pollen sterility, but was independent of *EFS*. It is worth emphasizing that *S24*-induced sterility was not affected by the *S35* genotype (comparison between #9 and #15 in Figure 3), as previously shown (Kubo *et al.* 2008).

### Genetic dissection of the S24 region and an activator of S35

**Figure 4:**
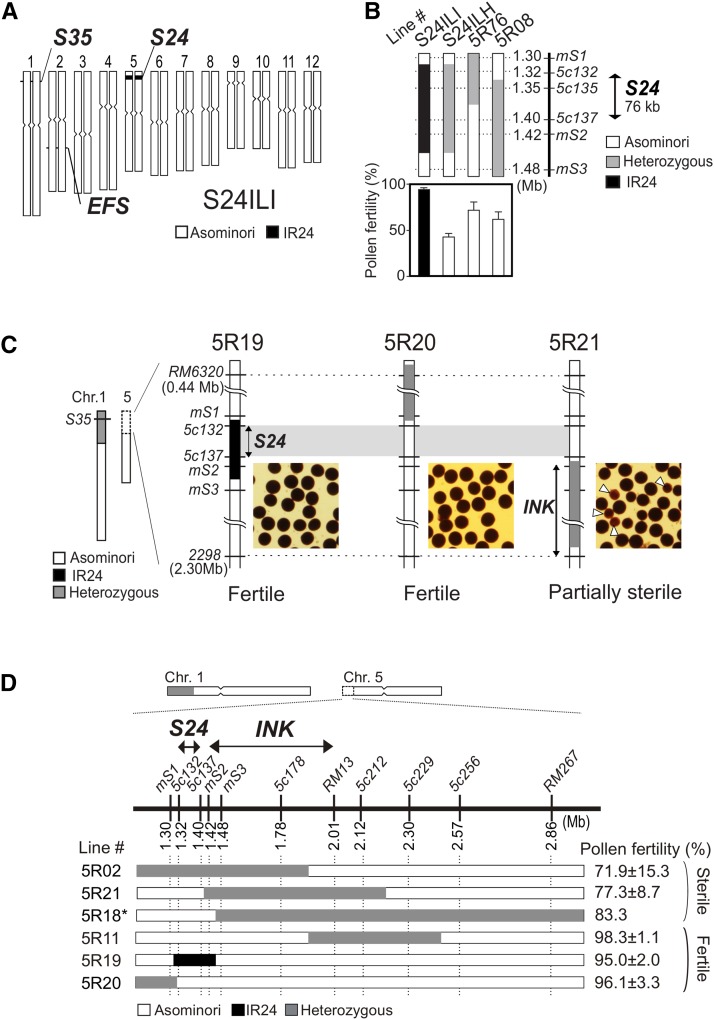
Identification and localization of the *S35*-activator. (A) Graphical genotype of the near-isogenic line S24ILI. S24ILI was homozygous for *S24* and carried a very small IR24 segment of less than 181-kb (*mS1*–*mS3*, see also Figure 4B) within a uniform Asominori background. (B) Pollen phenotype of the most informative recombinants and the NILs S24ILI/H. These lines carried Asominori homozygous alleles for both the *EFS* and *S35* loci, representing the pollen sterility dependent exclusively on *S24*. Bars show the mean (%) with SD (*n* = 4–9). (C) Pollen fertility of a set of substitution lines covering the *S24* region on chromosome 5. All the lines were heterozygous for *S35*. The white arrows in the photo indicate degenerated pollen grains. (D) Chromosome position of the *S35*-activator *INK* locus on chromosome 5. The diagram shows the recombination breakpoints of the obtained plants/lines. All plants/lines carried heterozygous alleles for *S35* to evaluate the effect of the recombinant segment on the *S35* phenotype. The pollen phenotype of each line was determined using selfed progeny (BC_4_F_3_) of the recombinant individuals (BC_4_F_2_) excluding 5R18. 5R18 was a single BC_5_F_1_ plant derived from a backcross of the recombinant BC_4_F_2_ plant carrying homozygous *S35-i* alleles. The 5R02 line had the *Efs-i* homozygous genotype that prevented the influence of heterozygous *S24* on pollen fertility. The mean (%) ± SD (*n* = 3–10) are shown on the right.

**Figure 5:**
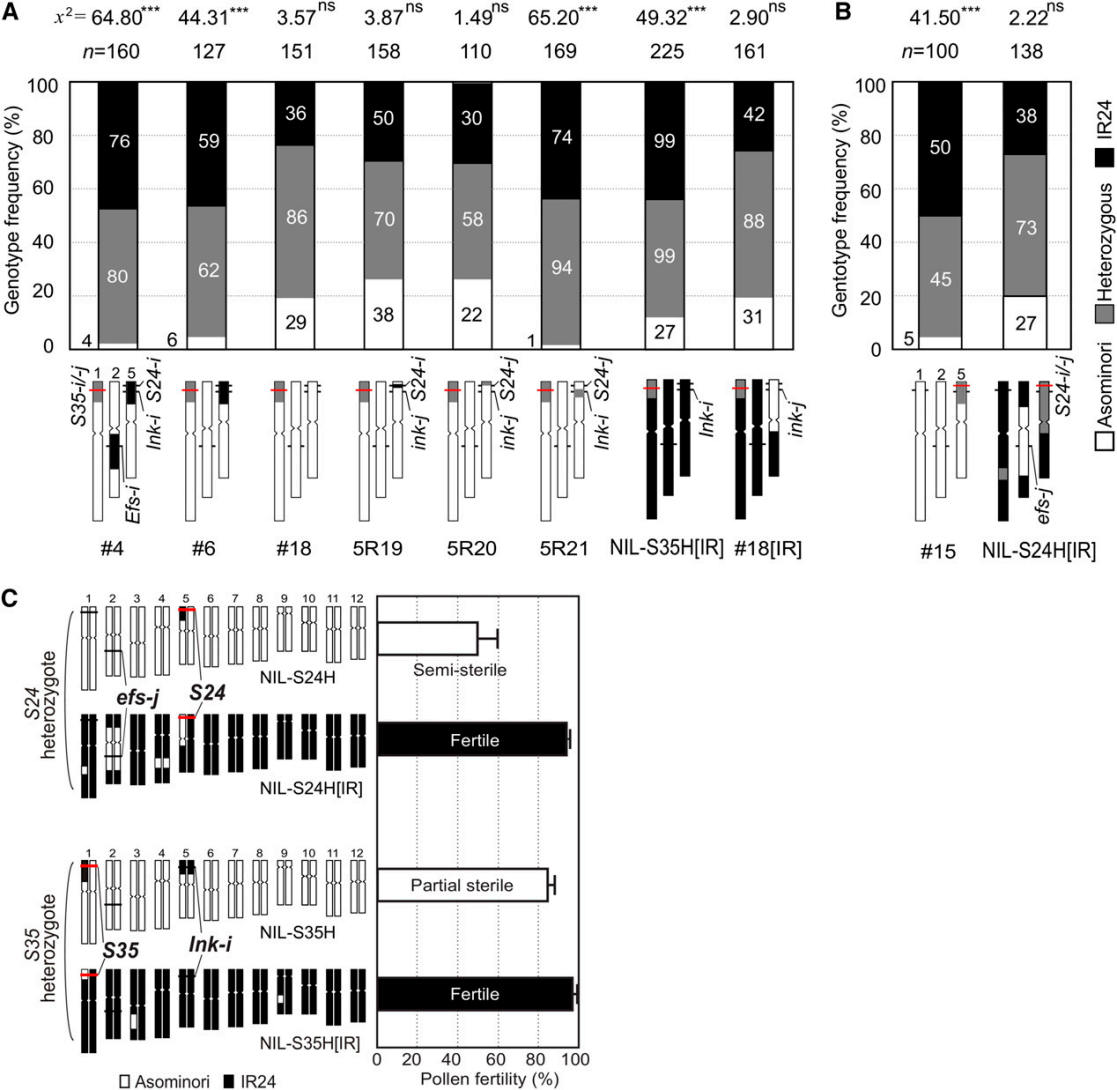
Effect of the genetic background on pollen sterility. (A), (B) Frequencies of the *S35* and *S24* genotypes in the selfed progeny of the NILs with reciprocal genetic backgrounds. The stacked bar chart represents the frequency of the *S35* genotype (A) and the *S24* genotype (B) in the selfed progeny of the heterozygotes. Population size and chi-square values for the deviation from 1:2:1 are shown on the top. The numbers of plants in each genotype (black, IR24 homozygote; gray, heterozygote; white, Asominori homozygote) are shown inside the bars. The parental genotype is shown at the bottom. DNA markers *1c350* or *1c305* (for *S35*), and *mS2* (for *S24*) were used for genotyping (the red line on chromosomes). The plant line ID is identical to that shown in Figure 3, Figure 4, and Figure 6. Parental plants #4, #6, #15, and 5R21 had sterile pollen. All other plants had fertile pollen. ** *P* < 0.01; *** *P* < 0.001; ns, not significant. (C) Graphical genotype (left) and pollen fertility (right) of the NILs that carried heterozygous segments harboring the *S24* or *S35* locus in either the Asominori or the IR24 genetic backgrounds. The NILs carried the appropriate genotype for the interacting partner gene (*efs-j/efs-j* for the *S24* activation, or *Ink-i/Ink-i* for the *S35* activation). Bars show the mean (%) with SD (*n* = 5).

For further characterization of *S24* as the pollen killer by itself and as the partner of *S35*, we genetically dissected the *S24* region using a segregating population (*n* = 485). We obtained a NIL of *S24* (called S24ILH/I) carrying a very small segment (181-kb) near the *S24* region with an otherwise uniform Asominori genetic background (Figure 4, A and B). The heterozygous segment of S24ILH was able to induce semisterility (43.1%) in the pollen, indicating that the small segment contained the *S24* locus (Figure 4B). We next reexamined the *S24–S35* interaction by analyzing the F_2_ and F_3_ individuals derived from a cross of S24ILI × NIL-S35al. From the self-pollinated progeny of an F_1_ plant, a plant homozygous for *S24-i*, and heterozygous for *S35*, was selected, and the pollen phenotype of its selfed progeny carrying the same parental genotype (5R19) was investigated (Figure S1B). If interaction between *S24* and *S35* occurred, the 5R19 would have sterile pollen due to the heterozygous *S35-i*/*S35-j* genotype. In contrast, the results indicated that 5R19 plants had good pollen fertility (95.0% fertility) (Figure 4, C and D), and the segregation pattern of *S35* fit a 1:2:1 ratio in the selfed progeny (Figure 5A). These results indicated that *S35* does not interact with *S24*, but rather with another locus linked to *S24*. In a similar way, we investigated the effects of other nearby segments on *S35*-dependent pollen sterility. A distal segment adjacent to the *S24* locus had no effect on pollen fertility (5R20 in Figure 4, C and D, and Figure 5A). On the other hand, another centromere-side segment resulted in partial sterility for pollen due to *S35* (5R21, 77.3% in Figure 4, C and D) and selective abortion of the *S35-j* gamete in the selfed progeny (χ^2^_1:2:1_ = 65.20, *P* < 0.001, Figure 5A). Thus, we were able to confirm that not *S24* but the *S24*-linked gene is essential for *S35* activation. This finding was consistent with the aforementioned result that *EFS* did not suppress *S35* and *S24*-induced pollen semisterility regardless of the *S35* genotype. The newly identified *S35*-activator locus was designated as *incentive for killing pollen* (*INK*). We next genetically dissected *INK* using 485 individuals. The *INK* locus was localized within a 592-kb region between marker loci *mS2* and *RM13* (Figure 4D). These results conclusively showed that the dominant *Ink-i* gene located close to *S24* was required to induce *S35*-dependent pollen sterility.

### Epistasis involving multiple genes controls pollen killers

**Figure 6:**
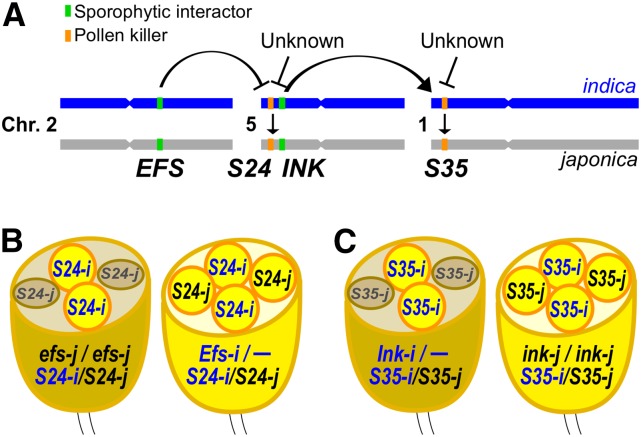
Diagram showing the genetic interactions between hybrid male sterility loci. (A) Schematic representation of the chromosome position and genetic interactions of hybrid male sterility genes. Arrows and bars represent positive and negative regulation, respectively. *Indica* has the killer alleles for the *S24* and *S35* loci (*S24-i* and *S35-i*), and these genes kill the male gamete bearing the *japonica* alleles in heterozygous hybrid progeny. Activation of these pollen killer genes is dependent on the sporophytic factors *EFS* for *S24* (B) and *INK* for *S35* (C). Additional unknown suppressors/modifiers for *S24* and *S35* exist within the *indica* genome.

As described above, we genetically dissected the *S35* and *S24* genes in the *japonica* genetic background. Through this analysis, we obtained clear evidence for two independent genetic pathways, *EFS–S24* and *INK–S35*. The question then arises as to whether these genes comprise the full set of genes involved in the pollen killer system. To address this question, we examined another set of NILs with an *indica* genetic background that have a nearly opposite genomic constitution. We developed two NILs, called NIL-S24H[IR] and NIL-S35H[IR] that carried *S24* or *S35* heterozygous alleles with the *indica* genetic background, respectively. Unlike the *japonica* background populations, pollen semisterility due to heterozygous *S24* did not occur in the IR24 genetic background (NIL-S24H[IR] in Figure 5C). Consistent with this normal pollen phenotype, a normal Mendelian segregation of *S24* was observed in the IR24 genetic background population (χ^2^_1:2:1_ = 2.22, *P* = 0.33) (Figure 5B). Also, the pollen fertility of the *S35-i/S35-j* heterozygotes was not significantly reduced in the IR24 genetic background (NIL-S35H[IR] in Figure 5C). Despite the fertile pollen phenotype, a reduced transmission of the *S35-j* allele was observed in the selfed progeny of NIL-S35H[IR] (Figure 5A). The frequency of the *S35-j* homozygote was slightly recovered in the IR24 genetic background (27/225 = 12.0%, in NIL-S35H[IR]) compared with that in the *japonica* background (2.5–4.7% in #4 and #6). Interestingly, the *S35* heterozygote with recessive homozygous *ink-j* alleles (*S35* held inactive) had a higher level of *S35-j* transmission {the *S35-j* homozygote emerged at 19.3% (31/161) frequency in the selfed progeny of #18[IR], Figure 5A}. Therefore, we considered that the effect of *S35* was slightly diminished, but *S35* still remained as a causal agent in the defective fertilization of *S35-j* pollen in the IR24 genetic background, even though no remarkable visible defects were found in their pollen grains when stained with the I_2_-KI solution. Genotype frequency data of a recombinant inbred (RI) population reported by Huang *et al.* (2009) supported these results. In the RI population derived from the cross between Nipponbare (*japonica*) × 93-11 (*indica*), the genotype frequency distribution peaked at 3.1 Mb on chromosome 1, where *S35* is located (Huang *et al.* 2009). The *japonica* homozygotes emerged at a reduced frequency [II:JJ = 115:33 (22%), the expected Mendelian ratio is 1:1 (50%), in Figure S3A), which was slightly higher than our data based on the *japonica* background populations [the expected value was estimated as II:JJ = 132:16 (11%) when the transmission frequency of the *indica S35* allele (*k*_i_ = 0.89, Table 1) was applied to the RI population]. Equal segregation of *S24* in the RI population was also in good agreement with our data obtained from NIL-S24[IR]. In total, these results indicated that *S35* was active in the *indica* background population as well as in the F_1_ hybrids, and also suggested that some minor modifier(s) existed in the IR24 genome. Furthermore, our results indicated that the action of *S24* was repressed by several suppressors encoded by the IR24 genome, including *EFS* (Figure 6A).

## Discussion

### Genetic mechanism of the pollen killer system

Although we hypothesized that *S35* caused pollen sterility via interaction with the *S24* locus (Kubo *et al.* 2008), the present study revealed that not *S24*, but a tightly linked locus *INK*, activates *S35* to cause pollen sterility. The *indica Ink-i* allele dominantly activated *S35*, and, conversely, *S35* had no harmful effect on reproductive or vegetative development in *japonica* homozygous for *ink-j/ink-j*. The *Ink-i* allele did not have a considerable effect on the activity of *S24* because *S24* caused pollen sterility without *Ink-i*. The independence of *S24* and *S35* was consistent with the result that the *S24*-suppressor *Efs-i* did not restore pollen sterility caused by *S35*. Consequently, we concluded that *INK–S35* and *EFS–S24* are independent pollen killer systems, rather than a single pathway of *EFS–S24–S35* (Figure 6B). In addition to these genes, the present study indicated the presence of additional modifier/suppressor(s) in the genetic background. A few minor modifiers for *S35*, and a major suppressor for *S24*, were suggested as the causes of phenotypic changes in the *indica* background. These findings suggest that the plant gamete killer system is regulated by multiple genetic networks involving linked and unlinked multiple genes with major and minor effects.

### Candidate genes and putative functions of S35 and INK

In this study, the *S35* locus was delimited within a 3.00–3.12 Mb region of chromosome 1 that contained many putative protein-coding genes. Within this chromosomal region (3.04–3.11 Mb), a hybrid male sterility locus *S-d* was found by using other *indica/japonica* cross combinations (Li *et al.* 2008). Since *S35* and *S-d* were located within the same narrow interval, these are hypothesized to be identical genes. Allelic differentiation of *S24* and *S35* seemed to contribute significantly to pollen sterility in the *indica-japonica* cross because both loci were found in different cross combinations of the *indica* and *japonica* varieties (Wang *et al.* 2006; Li *et al.* 2006, 2008). It is of great interest to understand the function of these genes in the parental species, as well as the harmful effects in the heterozygous hybrids. The *S35* locus caused developmental defects after the late uninucleate microspore stage (Figure 2A), suggesting that the causal gene is expressed and functions around this stage. The uninucleate stage is followed by two mitotic cell divisions in which many different genes are expressed, and dynamic changes in expression patterns occur, especially during the bicellular–tricellular transition (Fujita *et al.* 2010). Based on the expression profiles during male gametogenesis in rice, we found three candidate genes for *S35* whose expression increased or changed significantly near the uninucleate stage (*i.e.*, Cys-rich domain protein/LOC_Os01g06460, fasciclin-like arabinogalactan-protein/LOC_Os01g06580, and zinc finger C3HC4 type domain protein/LOC_Os01g06590 in Figure S2). Protein sequence polymorphisms were found in the Cys-rich domain protein and the fasciclin-like arabinogalactan-protein based on the *indica* variety 93-11 (data not shown). Therefore, these two genes are strong candidates for *S35/S-d*. It is noteworthy that a rice mutant study has revealed that a fasciclin glycoprotein (*Microspore and Tapetum Regulator 1*, *MTR1*) is essential for male gametogenesis in rice (Tan *et al.* 2012). As is the case with the *S-d* mapping study (*N* = 2160), no recombination events were found within the 122-kb candidate interval of the *S35* locus, suggesting a suppression of recombination within this region (a total of about 5000 individuals were screened for recombination events in the *S35/S-d* region in these two studies). A large chromosome insertion (∼36 kb) and many SNPs were found in the *S35/S-d* candidate region of the *indica* variety 93-11 (data not shown).

The *INK* locus was localized within a 592-kb region where 72 putative protein-encoding genes, and 20 transposable elements were found based on the Nipponbare genome sequence. No identical or similar genomic fragments were found between the *S35* and *INK* candidate regions. The *INK–S35* interaction fits well with an “epistasis-based allelic interaction model” that hypothesizes that the pollen killer system functions by interaction between sporophytic and gametophytic genes, as proposed previously (Kubo *et al.* 2011). In the case of *INK–S35*, *INK* is the sporophytic factor (Figure 6C). Therefore, the *INK* gene is predicted to be expressed in sporopytic tissues such as tapetal and anther wall layers. Since tapetal cells supply the major components of the pollen wall and nutrition requisite for pollen development, interaction between tapetal cells and microspore cells is essential for normal pollen development (Tan *et al.* 2012). Thus, the causal molecule encoded by *INK* may be involved in such a cooperative interaction between sporophytic and gametophytic gene products. This situation resembles the *SaF-SaM* male sterility system (Long *et al.* 2008). In that system, two genes, *SaF* and *SaM*, interact with each other to cause male sterility in an *indica/japonica* rice hybrid. The *indica SaF*^+^ allele acts sporophytically, whereas the *japonica SaM*^−^ allele acts as the gametophytic determinant for the selective pollen killing. A physical protein interaction of SaF^+^ with SaM^−^ but not with SaM^+^ was demonstrated (Long *et al.* 2008). Further cloning analyses will provide valuable information on the molecular interaction of *INK–S35*, and the molecular pathway underlying hybrid male sterility.

### Properties of the S24 and S35 gene blocks

In this study, we examined the effect of the *S24*–*INK* gene block on chromosome 5, focusing on pollen sterility. Interestingly, a number of studies have detected a major QTL for seed set percentage on the *S24*–*INK* region in several *indica/japonica* populations (Li *et al.* 1997; Wang *et al.* 1998; Chin *et al.* 2011). More recently, Zhao *et al.* (2007) identified a hybrid female sterility locus *S31* at 1.29–1.48 Mb within the *S24–INK* region by using the same Asominori-IR24 CSSLs. We expect that *S31* will facilitate a transmission advantage of the *indica S24–INK* haplotype through the female gametes. Intriguingly, however, no remarkable phenotype for the *S31* gene has been found in our growth conditions (Fukuoka and Mishima in Japan), although *S31* caused partial female sterility (about 65%) when the same CSSLs were grown in Nanjing, China (Zhao *et al.* 2007). This inconsistent gene action may be explained by differences in the environments where the experimental populations were grown. If so, this observation means that *S31* is strongly influenced by environment and can enhance the transmission of the *S24–S31–INK* gene complex under certain conditions. In terms of pollen sterility due to *S24* and *S35*, some variations were found within and among the NIL populations (Figure 1, Figure 3, and Figure 4). Phenotypic variation can result from gene–gene and gene–environment interactions. Linked or unlinked modifier gene(s) located in retained segments in the genetic background is a possible genetic element for phenotypic variation. A straightforward example of this can be found in the difference in pollen fertility among NILs for *S24* (Figure 4B). The pollen fertility of S24ILH with the smallest segment harboring the *S24* locus was lower than that of other recombinant lines (5R76 and 5R08), implying the presence of linked minor gene(s) for male-fertility restoration. Also, the *S35* locus showed some phenotypic differences (65–85%) between the NIL populations (Figure 1B and Figure 3). Environmental effects for pollen sterility due to *S35* were seen under different growth conditions [54.3 ± 7.3% in Fukuoka in Kubo *et al.* (2008); 84.2 ± 4.1% in Mishima in this study]. This environmental susceptibility may be a common feature of genes conferring male sterility in plants (Sakata *et al.* 2010; Ding *et al.* 2012; Wang *et al.* 2013; Kubo *et al.* 2016).

### The impact of pollen killer genes on the genetic characteristics of hybrid offspring

The actions of the *S35* and *S24* killers were exclusively associated with their heterozygotes. Homozygotes for either alleles did not show any notable phenotype in reproductive development or vegetative growth, excluding phenotypic changes by other linked genes. Both pollen sterile *S24*-heterozygotes and *S35*-heterozygotes exhibited nearly full seed set (93.3% ± 3.2, *n* = 10, in S24ILH; 88.4% ± 0.7, *n* = 3, in NIL-S35H), suggesting that these pollen killers have a transmission advantage for increasing their frequencies with no or a small effect on individual fecundity, at least in the *japonica* genetic background. But, as it is, such a strong transmission advantage cannot directly apply to early segregating generations of the *indica-japonica* hybrid progeny. In view of the results obtained in the RI and the *indica* background populations, *S35-i* may weaken its function of eliminating *S35-j* (*japonica* allele) but may still keep the killing action to some degree. In contrast, *S24* contributed little to the reduction of pollen fertility in the F_1_ generation. The action of *S24* is substantially suppressed by *Efs-i* and other suppressor(s).

Using *indica-japonica* hybrid populations, Hiko-Ichi Oka demonstrated that recombination of two independent genes tended to be restricted as if they were linked (referred to as pseudo-linkage or quasi-linkage) (Oka 1988). The same tendency was also observed in *O. sativa* × *Oryza glaberrima* (Sano *et al.* 1980). An example is the inheritance of *Phenol reaction* (*Ph*) and *Chromogen for anthocyanin* (*C*) located on separate chromosomes. The F_2_ population of *indica* (*Ph–c*) × *japonica* (*ph–C*) had excessive numbers of the parental genotypes (*Ph–c* and *ph–C*), and reduced numbers of recombined genotypes (*Ph–C* and *ph–c*) (Oka 1988). In this study, Oka pointed out that a gene set for duplicate gametic lethals is one possible cause for the pseudo-linkage of the phenotypic markers. Theoretically, the *INK–S35* interaction could also partially contribute to pseudo-linkage. The pollen killer *S35-i* (*indica* allele) increases its own transmission with the existence of the *indica Ink-i* allele through the male gamete, resulting in a high frequency of the *indica–indica* pair for the *S35* and *INK* alleles (*S35-i–Ink-i*) and a reduction of the recombinant pair of the *japonica–indica* alleles (*S35-j–Ink-i*). By this means through male gametes, the parental set of different chromosomes (chromosomal segments) will gradually increase their frequency in the population. Some of this tendency was observed in the two RI populations of Nipponbare × 93-11 (Huang *et al.* 2009), and Asominori × IR24 (Tsunematsu *et al.* 1996) (Figure S3B). Thus, several types of hybrid sterility genes may contribute to pseudo-linkage of many chromosomal regions because more than 40 hybrid sterility genes are dispersed throughout the rice genome.

### Evolutionary implications of pollen killers

In the traditional triallelic model, both the gamete killer and the sensitive alleles can arise from the original neutral allele without a reduction in fitness, and consequently become fixed in diverging populations. Recent studies of the *Sa* and *S5* loci have afforded tangible evidence of the molecular and the evolutionary mechanisms (Long *et al.* 2008; Yang *et al.* 2012). These loci contain multiple linked genes. Presumably, sequential mutations within these genes occur in either or both lineages, and these divergent haplotypes negatively interact, resulting in gamete dysfunction in the heterozygotes (Ouyang and Zhang 2013). This sequential divergence theory could be applicable to the pollen killer systems by *S35* and *S24*, despite the differences in physical positions of the interacting partner genes. Pollen killing can occur only if the pollen killers are paired with particular alleles at other interacting loci in the hybrid offspring. Such a polygenic system allows the accumulation of mutations at related loci without a significant fitness cost during evolution. In fact, an increasing number of studies have suggested that a gradual accumulation of multiple epistatic genes with no, or a small, individual effect may play important roles in the development of reproductive isolation in initial speciation (Cabot *et al.* 1994; Orr 1995; Kao *et al.* 2010; Kubo *et al.* 2016). In addition, the existence of third neutral alleles for *S24* and *S35* have been reported in studies using numerous cultivars and the wild progenitor *Oryza rufipogon* (Wang *et al.* 2006; Shi *et al.* 2009; Liu *et al.* 2011). This observation implies that these pollen killer loci may contain tightly linked multiple genes like *S5* and *Sa*, or may be pure trialleles of a single gene. Our study demonstrated the impact of the gene complex *S24–S31–INK* on hybrid sterility between *indica* and *japonica*. Plant species have evolved mainly through repeated duplication of an ancestral genome, chromosome and/or genes. Redundancy of gene sets in plant genomes is important as a prerequisite for diversification of genes adapting to environmental changes. Furthermore, most plants have the ability to reproduce asexually and via autogamy. Therefore, coadapted gene complexes can be maintained over many generations without breakdown by reproductive meiosis and meiotic recombination. These genomic and reproductive characteristics of plants should facilitate the formation of polymorphic gene complexes for reproductive isolation (Dempewolf *et al.* 2012).

Plant genetic studies have traditionally focused on gamete killer loci. Even if interacting partner genes exist in a genetic background, these partners would have largely gone unnoticed. Though much still remains to be elucidated, it is clear that many genetic factors, and a genetic network, play a pivotal role in the plant gamete killer system. Tracking down the genes one by one can, therefore, shed further light on the molecular and evolutionary mechanisms of gamete killer systems. Knowledge of the chromosomal locations and the nature of gamete killer loci will aid the design of efficient marker-assisted schemes for crop breeding.

## Supplementary Material

Supporting Information
