# Supplementary material for: Pollen Killer Gene S35 Function Requires Interaction with an Activator That Maps Close to S24, Another Pollen Killer Gene in Rice
Source: G3 (Bethesda). 2016 Mar 21;6(5):1459–68. doi: 10.1534/g3.116.027573 (PMC4856096; doi:10.1534/g3.116.027573)
Supplement: Supporting Information [file supp_6_5_1459__index.html]

Pollen Killer Gene S35 Function Requires Interaction with an Activator That Maps Close to S24, Another Pollen Killer Gene in Rice — Pollen Killer Gene S35 Function Requires Interaction with an Activator That Maps Close to S24, Another Pollen Killer Gene in Rice — Supporting Information 

# Pollen Killer Gene *S35* Function Requires Interaction with an Activator That Maps Close to *S24*, Another Pollen Killer Gene in Rice

## Supporting Materials for Kubo, Yoshimura, and Kurata, 2016

**Files in this Data Supplement:**

- Figure S1 - Crossing scheme of the near-isogenic lines (NILs) for the hybrid male sterility genes. (.pdf, 115 KB)
- Figure S2 - Expression patterns of the candidate genes for *S35* in rice tissues. (.pdf, 72 KB)
- Figure S3 - Genotype frequencies at pollen killer loci in recombinant inbred populations of *indica/japonica*. (.pdf, 105 KB)
- Table S1 - Primer sequences used in this study. (.pdf, 55 KB)
- Table S2 - Sequence annotation of the *S35* region. (.pdf, 75 KB)
