# Supplementary material for: Pollen Killer Gene S35 Function Requires Interaction with an Activator That Maps Close to S24, Another Pollen Killer Gene in Rice
Source: G3 (Bethesda). 2016 Mar 21;6(5):1459–68. doi: 10.1534/g3.116.027573 (PMC4856096; doi:10.1534/g3.116.027573)
Supplement: Supporting Information [file supp_g3.116.027573_FigureS1.pdf]

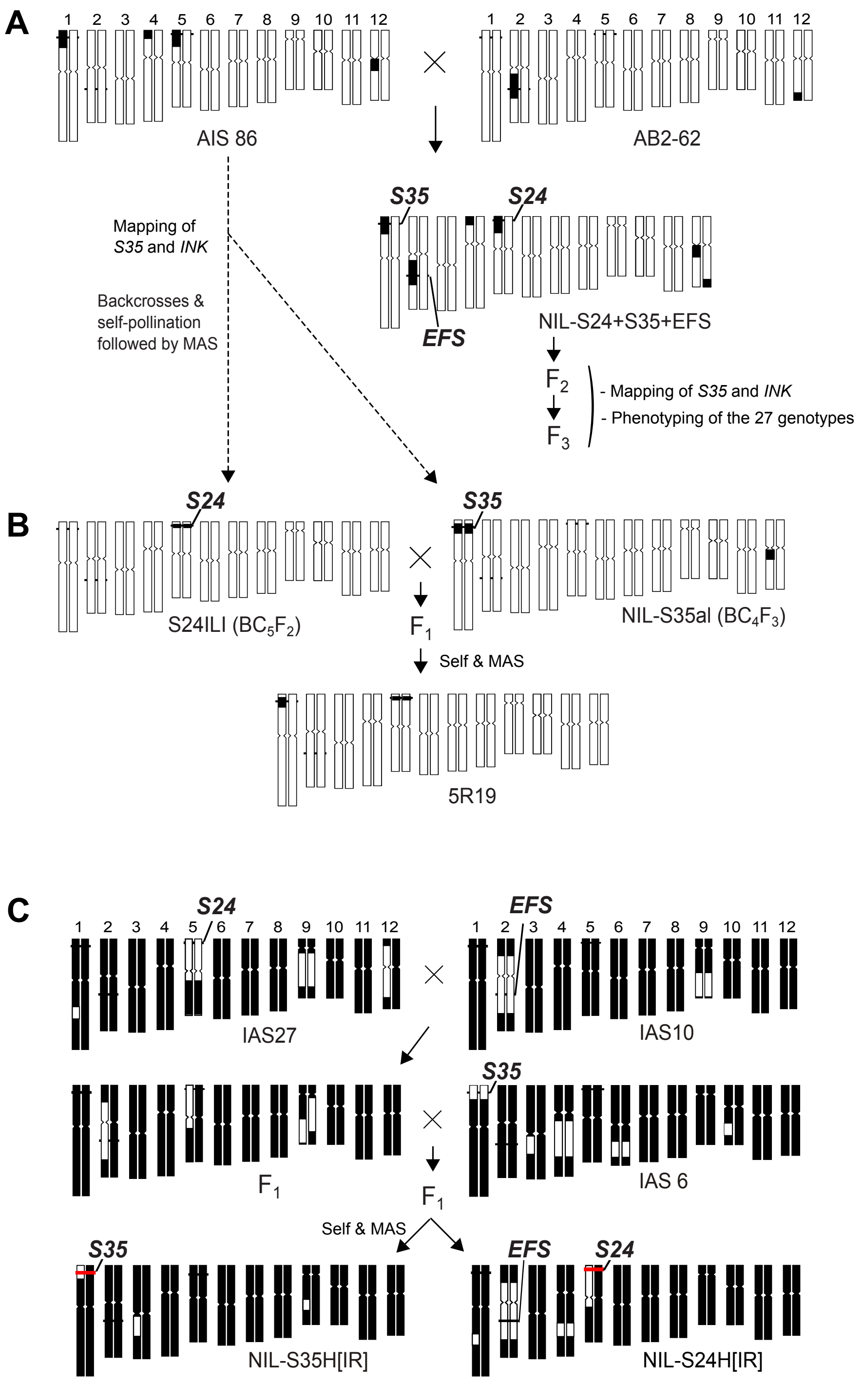

Figure S1. Crossing scheme of the near-isogenic lines (NILs) for the hybrid male sterility genes. All the experimental populations used in this study were derived from the two reciprocal CSSLs, AIS, IAS, and their sister lines that were reported previously (Kubo *et al*. 2002). IAS: Asominori CSSL with an IR24 background, AIS: IR24 CSSL with an Asominori background. The whole genome genotypes are referenced in Kubo *et al*. (2002). (A) A triple heterozygous NIL (NIL-S24+S35+Efs) was developed from a cross between AIS 86 (BC_3F_n) and AB2-62 (BC_3F_5). (B) Line 5R19 was selected from the selfed progeny of a F_1 plant between S24ILI and NIL-S35al. (C) The NILs with an *indica* genetic background were obtained from selfed progeny of the IAS 27/IAS 10//IAS 6 crosses. Only individuals heterozygous for *S24* or *S35* were selected and investigated for pollen fertility. Black bar: IR24 chromosome, White bar: Asominori chromosome, for A and B.
