# Supplementary material for: Pollen Killer Gene S35 Function Requires Interaction with an Activator That Maps Close to S24, Another Pollen Killer Gene in Rice
Source: G3 (Bethesda). 2016 Mar 21;6(5):1459–68. doi: 10.1534/g3.116.027573 (PMC4856096; doi:10.1534/g3.116.027573)
Supplement: Supporting Information [file supp_g3.116.027573_FigureS2.pdf]

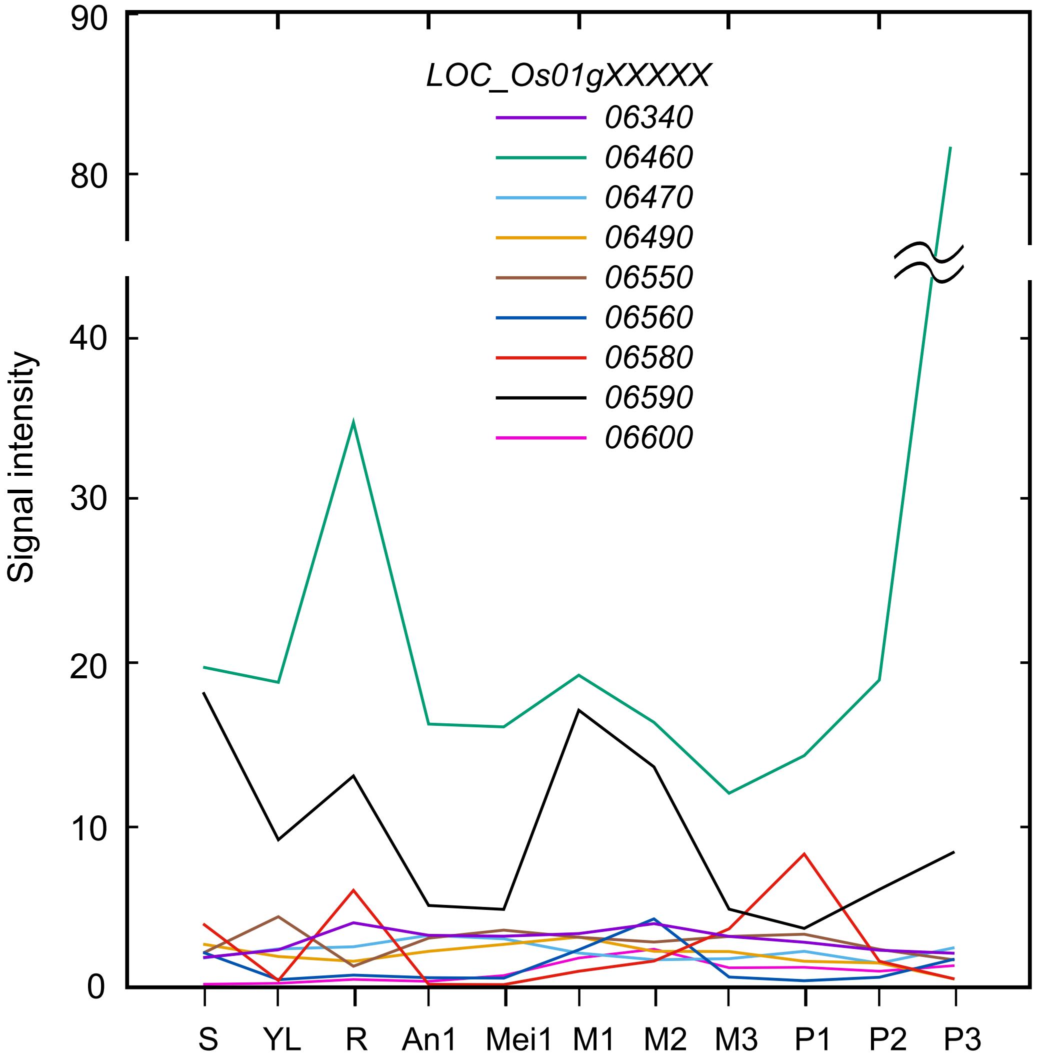

Figure S2. Expression patterns of the candidate genes for *S35* in rice tissues. These expression profiles were based on Affymetrix microarray data (GEO, GSE14304) previously reported by Fujita *et al*. (2010). The vertical axis represents normalized signal intensities as reported previously (Fujita *et al.* 2010). The horizontal axis represents selected rice tissues or developmental stages of anthers. S, 4-week-old shoot; YL, young leaf; R, root; An1–Mei1, anthers at the pre-meiotic stage; M1, at leptotene; M2, from zygotene to pachytene; M3, from diplotene to tetrad; P1, anther containing uninuclear pollen; P2, bicellular pollen; P3, tricellular mature pollen.
