# Supplementary material for: Pollen Killer Gene S35 Function Requires Interaction with an Activator That Maps Close to S24, Another Pollen Killer Gene in Rice
Source: G3 (Bethesda). 2016 Mar 21;6(5):1459–68. doi: 10.1534/g3.116.027573 (PMC4856096; doi:10.1534/g3.116.027573)
Supplement: Supporting Information [file supp_g3.116.027573_FigureS3.pdf]

A

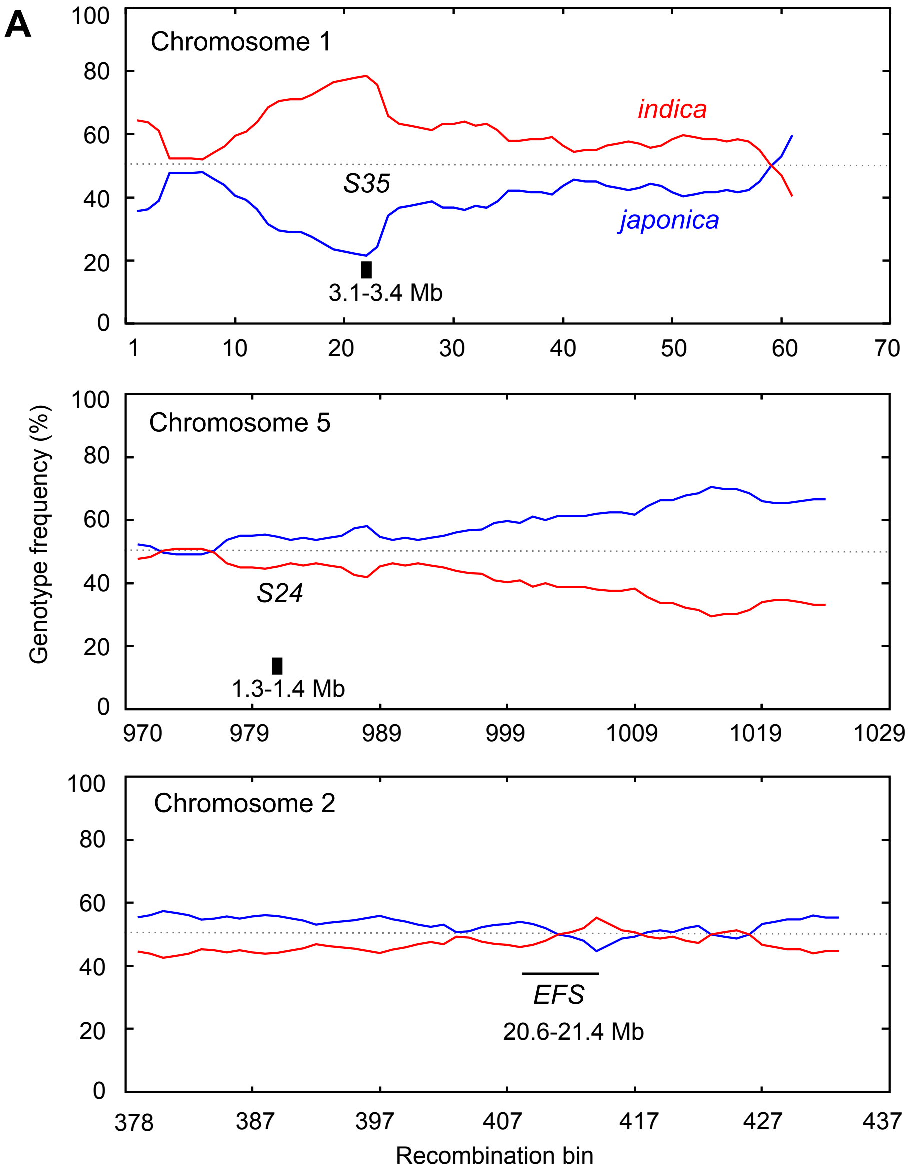

B

Nip x 93-11		*S35* II	*S35* JJ	total
INK	II	56	12	68
	JJ	59	21	80
total		115	33	148

x^2(1:1:1:1)=46.65, *P*<0.001

Aso x IR24		*S35* II	*S35* JJ	total
INK	II	30	7	37
	JJ	18	14	32
total		48	21	69

x^2(1:1:1:1)=16.16, *P*<0.01

Figure S3. Genotype frequencies at pollen killer loci in recombinant inbred populations of *indica/japonica*. (A) Genotype frequency distributions around the *S35*, *S24* and *EFS* loci in a recombinant inbred (RI) population of Nipponbare × 93-11. All individual lines of the RI population (N = 150 in F_{11} generation) were genotyped based on whole-genome resequencing data by Huang *et al*. (2009). The line charts are drawn based on a recombination bin map. The plotted region is 0–8.0 Mb (Bin ID 1–61) of chromosome 1, 0–8.2 Mb (Bin ID 970–1024) of chromosome 5, and 16.2–24.0 Mb (Bin 378–433) of chromosome 2. The *indica* and *japonica* homozygous genotypes are shown in red and blue, respectively. The expected genotype frequency (50%) is shown by a grey dotted line. A few RI lines heterozygous for the targeted loci were excluded from the evaluation of the genotype frequency. Positions of *S35*, *S24* and *EFS* loci are shown on the graph. (B) Genotypic frequencies of *S35* and *INK* in the two RI populations. The number in each genotype class was counted based on the genotype at recombination bin ID 21 (2.9–3.1 Mb, for *S35*) and 984 (1.6–1.9 Mb, for *INK*) in Nipponbare × 93-11 population, and RFLP loci *R1613* (3.4 Mb for *S35*) and *R3166* (1.9 Mb for *INK*) in Asominori × IR24 population. II: *indica* homozygote, JJ: *japonica* homozygote. By χ^2 tests (df = 3), the two RI populations differed significantly from Mendelian segregation (1 : 1 : 1 : 1).
