# Supplementary material for: Pollen Killer Gene S35 Function Requires Interaction with an Activator That Maps Close to S24, Another Pollen Killer Gene in Rice
Source: G3 (Bethesda). 2016 Mar 21;6(5):1459–68. doi: 10.1534/g3.116.027573 (PMC4856096; doi:10.1534/g3.116.027573)
Supplement: Supporting Information [file supp_g3.116.027573_TableS1.pdf]

Table S1 Primer sequences used in this study

Marker	Primer sequence (5'-3') Forward	Reverse	Chromosome	Chromosome position (bp)[a]
1c275	AGGCACAATGAGAAACAACAG	TTGATCCAACGGTTGATAACTCT	1	2,751,078
1c296	ATTCAGGGACAGCAAGATCG	CAGGATCCTCTCCCTCTCCT	1	2,961,120
1c300	GGTTGAGCACGAGGCAAA	CTGGTGATACGGTGATGGTG	1	3,003,020
1c305	CAGACTCTTCTCCGGTCAGG	GGCTAAGCCATGCTAACCAG	1	3,047,780
1c310	CGGGGAGAAGATAGGAGGAG	AAATACGAGAGCCCTTTCCA	1	3,105,116
1c312	ACATTGGGAGTACGAACGAA	TGGTGATGAAAAGGTGAGGA		3,122,703
1c314	GGATCGAGAAGCCAAAACAT	CGCACAACGCTTAGTCAAAA	1	3,140,791
1c318	ACTCAAAGTCACCGGGATTG	TTGCACCATGATACGTGAAAA	1	3,179,738
1c324	GGTGGTGATGGAAGAAGAGC	TCCGAGATAACCACCACCTC	1	3,244,005
1c340	CTGACCAACCCTACTTGAGTCT	GTTGATTGGGTCTTTGTCTCA	1	3,394,097
1c350	ATCACCATCCTCCTCCTCCT	CAAGGAAGGAGAAGCAACCA	1	3,505,825
2c2015[b]	TGTGGTGCTTGCGTTACTG	GGGGTGAAGAGATAGAATTGAAG	2	20,163,620
RM6320[d]	GAGCTGGACCTCCTCGACAC	CATGCATCACCGAATGAGTC	5	471,605
mS1	AAACAGTAAGGCTCACCAATTT	GAAGCAACTCTGGGGTCAAT	5	1,296,882
5c132	ACCATCCTCGTGGCCTGC	TAGCTGCAAAACTGGTGGTG	5	1,320,405
5c135[c]	GGGAAACCCCCAATCTATCT	AAAACGACCCGGCCATTCATCATA AAACTACTGC	5	1,346,315
5c137	CGGGATGAAAGTGGTGAGAT	GAGGAAGTGATCCGTTTCCA	5	1,370,242
mS2	GCATGGCATTTTCCAACTTT	ATGGCAGATGCATCAGAATG	5	1,419,360
mS3	TCTTGAAATCTCCTGCCTTC	TCAGACAATTCAGCCTCTCA	5	1,477,258
5c178	GAGCGAGCAACCAGCTAGAC	TAATCCGCCCTCTACATCCA	5	1,781,598
RM13[d]	TCCAACATGGCAAGAGAGAG	GGTGGCATTCGATTCCAG	5	2,011,188
5c212	GCCCCACAACTAATCCACAC	TATTCCTCCTGCTGGCCTTT	5	2,122,904
5c229	TTTCTATCATTGGCCAAATTCA	CAAAACCGACTAGGGGCATA	5	2,287,101
5c230	AAGACAACGGCAGTGTGATG	GTGGGCTAAGAAAAGGCTGT	5	2,298,638
5c256	TTGGAACTTTGGAAGGCAAG	TGATGCAGAACCACTCGAAG	5	2,567,550
RM267[d]	TGCAGACATAGAGAAGGAAGTG	AGCAACAGCACAACTTGATG	5	2,881,458

[a] The chromosome position was based on the Nipponbare genome sequence (IRGSP 1.0).

[b] 2c2015, a PCR marker tightly linked to *EFS* locus, was used for genotyping of the *EFS* region in MAS.

[c] 5c135 was used as a CAPS marker and needs *Pst* I digestion after PCR amplification.

[d] PCR markers previously reported in McCouch *et al.* 2002.
