# Supplementary material for: Pollen Killer Gene S35 Function Requires Interaction with an Activator That Maps Close to S24, Another Pollen Killer Gene in Rice
Source: G3 (Bethesda). 2016 Mar 21;6(5):1459–68. doi: 10.1534/g3.116.027573 (PMC4856096; doi:10.1534/g3.116.027573)
Supplement: Supporting Information [file supp_g3.116.027573_TableS2.pdf]

Table S2 Sequence annotation of the *S35* region

MSU locus ID	Transcript evidence *japonica*	Transcript evidence *indica*	Annotation	Reported phenotype by family members	Expression level in developing anther[a]	Microarray probe ID (Affymetrix)
LOC_Os01g06310	AK241045	CT834880	Glycine-rich cell wall structural protein precursor	Cytoplasmic male sterility in rice, Starch accumulation in anther wall cell in lily, exine formation in tomato[b,c,d]	low (< 1.0)	Os.6389.1.S1_at
LOC_Os01g06320	AK106969		MYB family transcription factor	Pollen and anther development[e,f]	low (< 1.0)	Os.31652.1.S1_at
LOC_Os01g06340	AK107993		DUF1110 protein		expressed	Os.31008.S1_at
LOC_Os01g06350			DUF1110 protein		-	N/A
LOC_Os01g06370			DUF1110 protein		low (< 1.0)	OsAffx.10555.1.S1_at
LOC_Os01g06380			Hypothetical protein		low (< 1.0)	OsAffx.22769.1.S1_at
LOC_Os01g06390			DUF1110 protein		low (< 1.0)	OsAffx.10553.1.S1_at
LOC_Os01g06400	AK108577		Hypothetical protein		low (< 1.0)	Os.30624.2.S1_
LOC_Os01g06410			DUF1110 protein		low (< 1.0)	OsAffx.23135.1.S1_at
LOC_Os01g06420			Hypothetical protein		low (< 1.0)	OsAffx.22993.1.S1_at
LOC_Os01g06430			DUF1110 protein		low (< 1.0)	OsAffx.23042.1.S1_at
LOC_Os01g06440	AK106766		Hypothetical protein		low (< 1.0)	Os.31782.1.S1
LOC_Os01g06450			Glycosyltransferase family 43 protein	Pollen wall formation[g]	low (< 1.0)	OsAffx.10552.2.S1_x
LOC_Os01g06454	AK121694		Heat shock protein DnaJ	Thermosenstive gametophyitc male sterility in Arabidopsis[h]	low (< 1.0)	Os.1460.2.S1
LOC_Os01g06460	AK072658		Uncharacterized Cys-rich domain containing protein	Pollen fertility in rice, pollen tube growth in tomato[i,j]	Highly expressed	Os.1460.1.S1
LOC_Os01g06470	AK103127		Importin alpha-like protein		expressed	Os.174.1.S1
N/A	AK109233	CT831329	Pentatricopeptide repeat containing protein	Fertility restorer for cytoplasmic male sterility[k]	low (< 1.0)	Os.56084.1.S1_at
LOC_Os01g06490	AK071183	CT833612	OsSCP1 - Putative Serine Carboxypeptidase homologue	Regulation of spikelet size by increasing cell number in rice[l]	expressed	Os.24975.1.S1
LOC_Os01g06500	AK063812	CT833350	Phloem 2-like A5		low (< 1.0)	Os.32936.1.S1
LOC_Os01g06510	AK241813		Arginyl-tRNA synthetase,		low (< 1.0)	Os.46028.1.S1
LOC_Os01g06520	AK070173		Verticillium wilt disease resistance protein		low (< 1.0)	Os.34779.1.S1
LOC_Os01g06540			PHD finger protein, DUF3594	Pollen tapetum development in rice and Arabidopsis[m,n]	low (< 1.0)	OsAffx.10938.1.S1_at
LOC_Os01g06550	AK066765		NF-X1-type zinc finger protein		expressed	Os.12454.1.S1.a_at
LOC_Os01g06560	AK106273		Transcription factor HBP-1b		expressed	Os.4770.1.S1
LOC_Os01g06570			Hypothetical protein		low (< 1.0)	OsAffx.23136.1.S1_at
LOC_Os01g06580	AK108308	CT831384	Fasciclin-domain arabinogalactan-protein	Tapetum and pollen development in rice[o]	expressed	Os.1437.1.Sl_at
LOC_Os01g06590	AK073728		Zinc finger, C3HC4 type domain containing protein		expressed	Os.1438.1.Sl_at
LOC_Os01g06600	AK100550		Glutaryl-CoA dehydrogenase, mitochondrial precursor		expressed	Os.1440.2.Sl_x_at

LOC_Os01g06450~LOC_Os01g06590 are located within the candidate region of the *S-d* locus.

[a] Gene expression was based on microarray data (GEO, GSE14304) previously reported by Fujita *et al*. (2010). " low (<1.0)" represents a signal intensity of less than 1.0 throughout all stages of anther development. Expression levels of the "expressed" genes are shown in Fig. S2.

[b] Hu J., Huang W., Huang Q., Qin X., Dan Z., Yao G., Zhu R., Zhu Y., 2013 The mechanism of ORFH79 suppression with the artificial restorer fertility gene Mt-GRP162. New Phytol. 199: 52–58.

[c] McNeil K. J., Smith A. G., 2010 A glycine-rich protein that facilitates exine formation during tomato pollen development. Planta 231: 793–808.

[d] Mousavi A., Hiratsuka R., Takase H., Hiratsuka K., Hotta Y., 1999 A novel glycine-rich protein is associated with starch grain accumulation during anther development. Plant Cell Physiol. 40: 406–16.

[e] Zhang H., Liang W., Yang X., Luo X., Jiang N., Ma H., Zhang D., 2010 Carbon starved anther encodes a MYB domain protein that regulates sugar partitioning required for rice pollen development. Plant Cell 22: 672–89.

[f] Xu Y., Iacuone S., Li S. F., Parish R. W., 2014 MYB80 homologues in Arabidopsis, cotton and Brassica: regulation and functional conservation in tapetal and pollen development. BMC Plant Biol. 14: 278.

[g] Moon S., Kim S.-R., Zhao G., Yi J., Yoo Y., Jin P., Lee S.-W., Jung K., Zhang D., An G., 2013 Rice glycosyltransferase1 encodes a glycosyltransferase essential for pollen wall formation. Plant Physiol. 161: 663–75.

[h] Yang K.-Z., Xia C., Liu X.-L., Dou X.-Y., Wang W., Chen L.-Q., Zhang X.-Q., Xie L.-F., He L., Ma X., Ye D., 2009 A mutation in Thermosensitive Male Sterile 1, encoding a heat shock protein with DnaJ and PDI domains, leads to thermosensitive gametophytic male sterility in Arabidopsis. Plant J. 57: 870–82.

[i] Hu L., Liang W., Yin C., Cui X., Zong J., Wang X., Hu J., Zhang D., 2011 Rice MADS3 regulates ROS homeostasis during late anther development. Plant Cell 23: 515–33.

[j] Tang W., Ezcurra I., Muschietti J., McCormick S., 2002 A cysteine-rich extracellular protein, LAT52, interacts with the extracellular domain of the pollen receptor kinase LePRK2. Plant Cell 14: 2277–87.

[k] Schmitz-Linneweber C., Small I., 2008 Pentatricopeptide repeat proteins: a socket set for organelle gene expression. Trends Plant Sci. 13: 663–70.

[l] Li Y., Fan C., Xing Y., Jiang Y., Luo L., Sun L., Shao D., Xu C., Li X., Xiao J., He Y., Zhang Q., 2011b Natural variation in GS5 plays an important role in regulating grain size and yield in rice. Nat. Genet. 43: 1266–9.

[m] Li H., Yuan Z., Vizcay-Barrena G., Yang C., Liang W., Zong J., Wilson Z. A., Zhang D., 2011a PERSISTENT TAPETAL CELL1 encodes a PHD-finger protein that is required for tapetal cell death and pollen development in rice. Plant Physiol. 156: 615–30.

[n] Ito T., Nagata N., Yoshiba Y., Ohme-Takagi M., Ma H., Shinozaki K., 2007 Arabidopsis MALE STERILITY1 encodes a PHD-type transcription factor and regulates pollen and tapetum development. Plant Cell 19: 3549–62.

[o] Tan H., Liang W., Hu J., Zhang D., 2012 MTR1 encodes a secretory fasciclin glycoprotein required for male reproductive development in rice. Dev. Cell 22: 1127–37.
